# Influence of Tooth Surface Pretreatment Procedure on Bond Strength of Self-Adhesive Cement—An In Vitro Study

**DOI:** 10.3390/dj14100653

**Published:** 2026-10-09

**Authors:** Sanita Grinberga, Evaggelia Papia, Janis Lungevics, Oskars Radzins, Janis Locs, Christel Larsson, Una Soboleva

**Affiliations:** 1 Department of General Dentistry, Rīga Stradiņš University, LV-1007 Riga, Latviauna.soboleva@rsu.lv (U.S.); 2 Institute of Stomatology, Rīga Stradiņš University, LV-1007 Riga, Latvia; 3 Faculty of Odontology, Malmö University, 205 06 Malmö, Sweden; evaggelia.papia@mau.se (E.P.); christel.larsson@mau.se (C.L.); 4 Faculty of Civil and Mechanical Engineering, Riga Technical University, LV-1048 Riga, Latvia; janis.lungevics@rtu.lv; 5 Baltic Biomaterials Centre of Excellence (BBCE), Riga Technical University, LV-1048 Riga, Latvia; janis.locs@rtu.lv; 6 Institute of Biomaterials and Bioengineering, Faculty of Natural Sciences and Technology, Riga Technical University, LV-1048 Riga, Latvia

**Keywords:** adhesive dentistry, bond strength, cleaning agent, self-adhesive resin cement, thermocycling, zirconia overlay

## Abstract

**Objectives**: Reliable bonding is essential for the longevity of zirconia restorations. This in vitro study evaluated whether tooth surface pretreatment with a cleaning agent influences the bond strength of zirconia overlays cemented with a self-adhesive resin cement under simulated aging conditions. **Methods**: Twenty-three intact third molars were collected. Three specimens were used for surface roughness measurements. Twenty specimens were prepared by exposing dentin surfaces and creating standardized retentive cavities. Micro-CT was performed to calculate precise dentin and enamel area proportions. Zirconia overlays were fabricated and cemented using a self-adhesive resin cement. Specimens were randomly assigned to one of two groups: a control group (*n* = 10) or a test group (*n* = 10), where a cleaning agent (Katana Cleaner) pretreatment was applied. After cementation, all specimens underwent 5000 thermocycles. Tensile bond strength was assessed, and failure modes were classified as adhesive or cohesive. **Results**: Linear regression analysis showed that dentin and enamel surface areas were the main factors associated with failure force, although the overall model fit was modest (adjusted R^2^ = 0.17, *p* = 0.1169). No statistically significant difference in failure rates was found between the pretreated group and control group (Fisher’s Exact test, *p* = 0.3034). No significant differences in failure modes were identified between the two groups. Roughness results show that etching with acid reduced the actual surface area by 1 to 1.5%, while further cleaning with Katana Cleaner led to further reduction by 0.1%. **Conclusions**: Bond strength of zirconia overlays cemented with self-adhesive resin cement appeared to depend primarily on substrate area and cavity retention. Additional tooth surface pretreatment with a cleaning agent did not significantly improve bond strength under the tested conditions.

## 1. Introduction

A central goal of restorative dentistry is the predictable reconstruction of lost tooth structure while preserving function, including phonation and esthetics [1,2]. The integration of digital workflow has enabled precise and efficient fabrication of indirect restorations such as inlays, onlays, and overlays [3,4,5]. Among these, overlays represent a minimally invasive approach that allows preservation of sound tooth structure while restoring function and appearance.

Cementation plays a decisive role in the long-term success of indirect restorations [6]. Conventional resin cements, including total-etch and self-etch systems, are considered highly reliable but require multiple technique-sensitive steps, such as etching, priming, and bonding, which increase chair time, the risk of procedural errors, and decrease ease of use [2,7]. These factors may limit their routine use, particularly among general practitioners.

Self-adhesive resin cements (SARCs) were introduced to simplify clinical workflows by combining adhesive and luting functions into a single material. Compared with multi-step systems, SARCs offer reduced chair time, fewer technique-sensitive steps, and potentially lower postoperative sensitivity, improving clinical efficiency [8,9,10].

From a materials science perspective, SARCs contain functional acidic monomers, particularly 10-methacryloyloxydecyl dihydrogen phosphate (MDP), which promotes chemical interaction with zirconia surfaces [11,12]. Recent formulations, such as Panavia SA Universal, combine MDP with silane chemistry to enable adhesion to multiple substrates without additional primers. In vitro studies have reported that certain SARCs can achieve bond strengths to zirconia comparable to conventional resin cements under controlled conditions [13].

Zirconia-based ceramics have gained increasing attention because of their high mechanical strength and wear resistance [14]. These properties make zirconia particularly suitable for patients exposed to high occlusal loads, including those with parafunctional habits such as bruxism. However, despite these advantages, zirconia restorations are not yet widely adopted in routine practice, partly due to ongoing concerns regarding reliable adhesive bonding to tooth structures [15]. This aspect is particular importance for overlays.

Surface pretreatment is another important factor affecting zirconia bonding. Mechanical roughening methods and MDP-containing primers can enhance micromechanical and chemical retention [16,17]. Despite considerable research on zirconia bonding, the role of surface pretreatment—particularly cleaning procedures—remains unclear. While zirconia cleaning agents have been shown to effectively remove contaminants and restore bonding potential without altering surface morphology, their direct influence on tooth surface characteristics and the resulting bond strength of self-adhesive resin cements is not yet fully understood [18,19,20]. Various cleaning and surface pretreatment protocols have been proposed to improve bonding performance, although their application may be limited by the substrate or by whether the procedure is intended for extraoral or intraoral use. Katana Cleaner (Kurary Noritake, Japan) is a relatively recent cleaning agent containing 10-MDP salt, developed for the cleaning of both dental hard tissues and prosthetic surfaces and intended for both intraoral and extraoral application. This versatility makes it of particular interest as a potential cleaning agent during the cementation of indirect zirconia restorations, where contamination of the bonding substrate may occur during clinical procedures [21,22].

Clinically, the simplified workflow offered by SARCs is highly appealing. Fewer procedural steps reduce technique sensitivity and treatment time, making adhesive cementation more feasible for general dentists [10,23]. Increased efficiency may also have economic benefits by optimizing clinical productivity while maintaining restoration quality and enhancing patient satisfaction.

Given the ongoing development of self-adhesive cements and the need to improve zirconia bonding protocols, further investigation is warranted to evaluate the effectiveness of surface pretreatment strategies and to verify manufacturer claims. Understanding these factors can support evidence-based clinical decision-making. Therefore, the aim of the present in vitro study was to evaluate the effect of tooth surface pretreatment on the bond strength of a self-adhesive resin cement to zirconia restoration. In addition, this study sought to assess whether the application of a cleaning agent alters the tooth surface in a way that may influence adhesive performance under simulated clinical aging conditions.

The research hypothesis of this in vitro study was that surface pretreatment with a cleaning agent prior to cementation of zirconia overlays using a self-adhesive resin cement improves bond strength to tooth substrates. Confirmation of this hypothesis would support a simplified, time-efficient clinical protocol without compromising adhesion quality, offering potential clinical and economic benefits.

## 2. Materials and Methods

### 2.1. Experimental Sample Description

**Figure 1:**
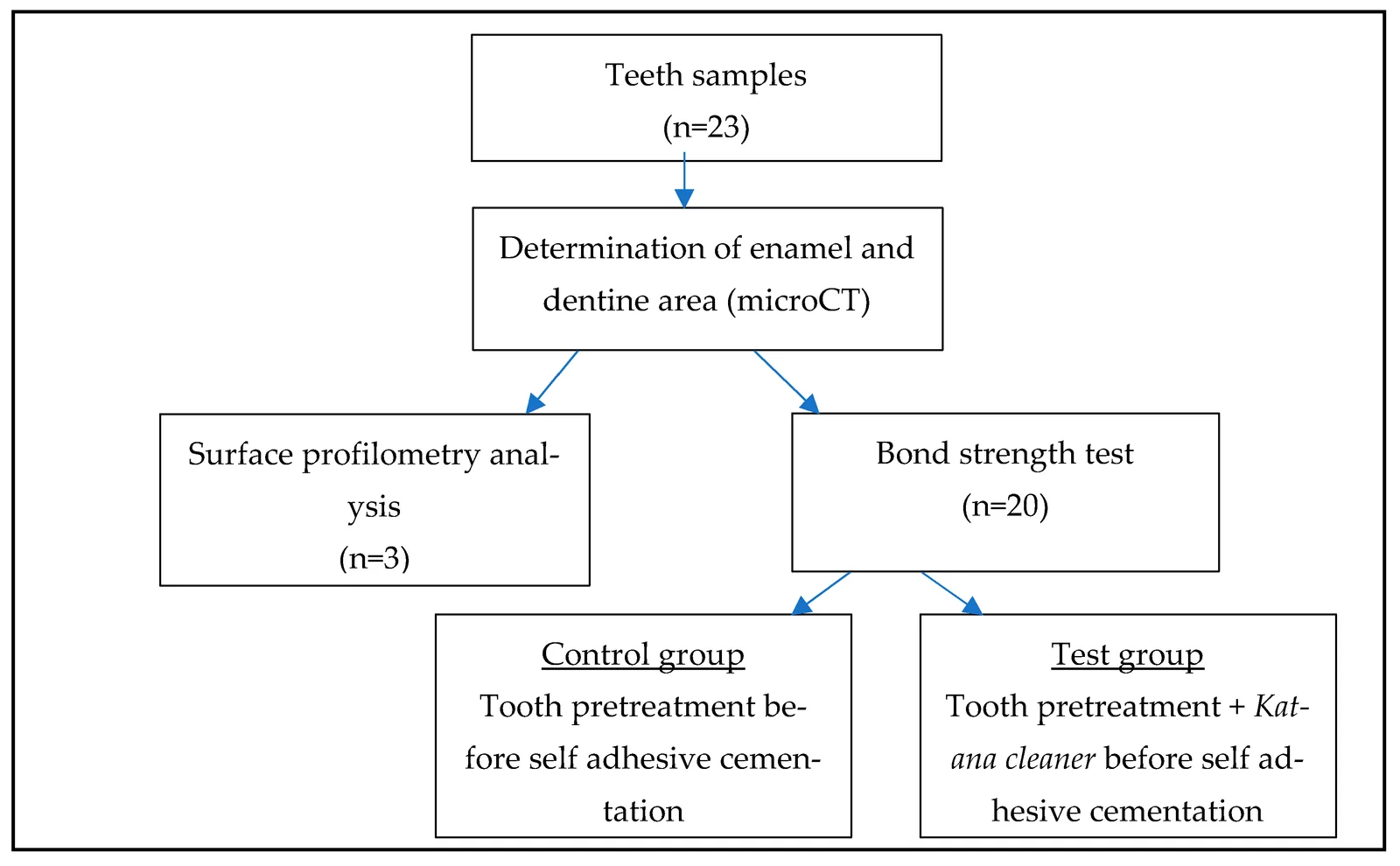
A flow chart of the study design.

The study received ethical approval from the Institutional Research Ethics Board No. 2-PĒK-4/88/2025. The sample included extracted third molars, which were removed between January 2025 and June 2025 for orthodontic reasons or due to unfavorable tooth position. The sample size was determined based on previously published in vitro studies, which commonly used similar sample sizes [5,24,25]. A total of 23 intact third molars free from caries, extensive restorations, abrasions, cracks, or hypocalcifications were collected. The teeth were cleaned of plaque, calculus, and periodontal tissue using an ultrasonic scaler (PiezoLED, KaVo Dental, manufactured by E.M.S. Electro Medical Systems S.A., Nyon, Switzerland). Following cleaning, the teeth were stored in physiological saline solution at 4 °C and kept refrigerated, with testing performed within six months after extraction. The experimental setup of the study is illustrated in Figure 1.

### 2.2. Tooth Sample Preparation for Surface Analysis and Bond Strength Test

**Figure 2:**
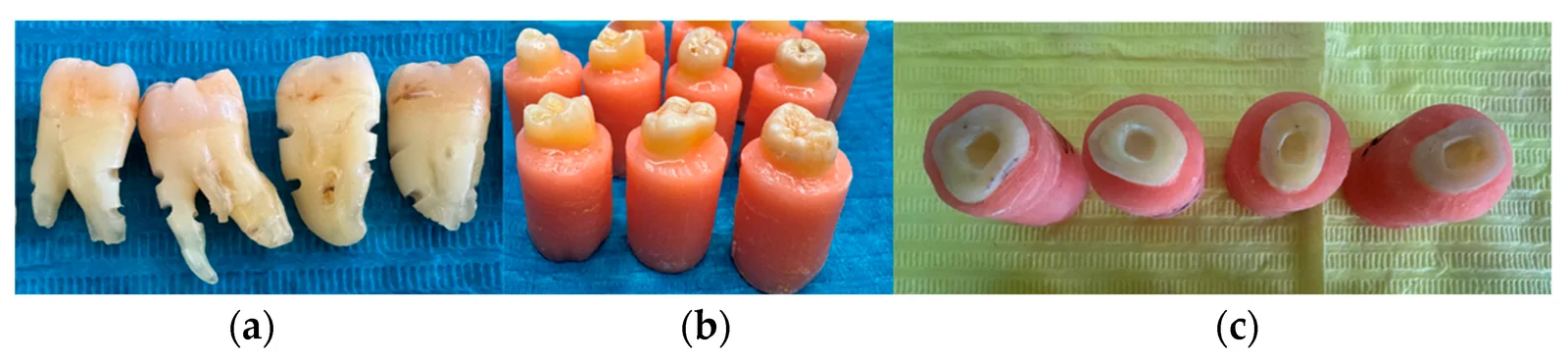
Tooth preparation steps: (**a**) Retention grooves on the root surface; (**b**) Specimens after PMMA curing; (**c**) Specimens prepared for bond strength test.

A total of 23 pre-selected teeth (*n* = 23) were embedded in chemically cured acrylic resin (PMMA SR Ivocap High Impact, Ivoclar Vivadent, Schaan, Liechtenstein) in cylindrical molds measuring 16 mm in diameter and 25 mm in height. Each tooth was positioned centrally and parallel to its long axis. To ensure adequate retention within the acrylic resin, depth grooves were prepared on the root surfaces before embedding. All specimens were prepared in the same manner by horizontally grinding the crown under continuous water cooling (FG Cylinder 881, Diamond Bur, 018, Edenta, Switzerland) to expose flat enamel and dentin surfaces above the pulp level. Following this standardized preparation, the specimens were divided into two groups. Following sectioning and cavity preparation, all specimens were visually inspected and examined with a dental probe to exclude caries, cracks, fractures, or other structural defects that could affect the bonding procedure or test results. Minor chronic fissure staining observed in some specimens was considered superficial pigmentation rather than caries based on visual and tactile examination. Three specimens (*n* = 3) remained without further cavity preparation and were used for surface roughness analysis. The remaining 20 specimens (*n* = 20) received a standardized 2 × 2 × 2 mm retentive box prepared in the dentin using a calibrated depth marker bur under water cooling (Depth Marker, TM20, 2.0, Edenta, Switzerland). The retentive cavity was prepared to prevent displacement of the restoration during cementation. (Depth Marker, TM20, 2,0, Edenta, Switzerland) (Figure 2a–c).

### 2.3. Tooth Sample Imaging and Contact Area Measurements (Micro-CT)

All specimens were scanned using a Rigaku CT Lab HX130 (CT Lab; Rigaku Corp., Tokyo, Japan) at 130 kV with a current of 60 µA, focus set to S, and using a Cu 0.1 mm filter. Scan acquisition settings were 25 mm FOV (field of view), short geometry, high-resolution scan mode, high image resolution (25.2 µm voxel size), and 4 min scan time.

**Figure 3:**
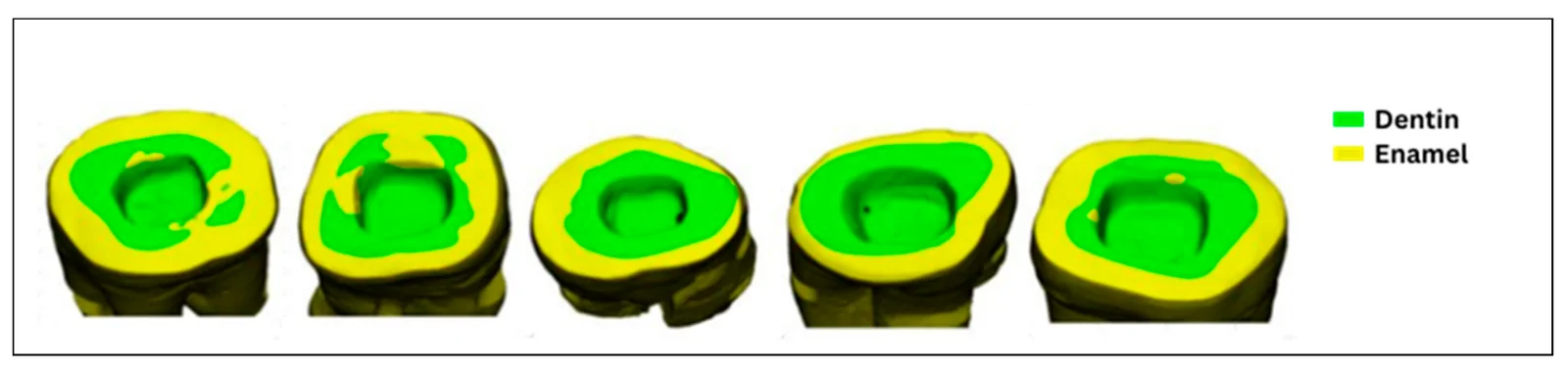
Enamel and dentine segmentation. Enamel is depicted in yellow and dentin in green.

All scanned specimens were then exported in uncompressed DICOM (Digital Imaging and Communications in Medicine) format and imported into Mimics (Materialise, Leuven, Belgium; version 27.0). A lower border segmentation was performed by setting the lower border thresholding value to 794 ± 13 and 896 ± 12 gray values for enamel and dentin areas, respectively, to gain the optimal signal-to-noise ratio on a per-sample basis. Subsequently, the segmentations were post-processed by implementing a wrapping (smallest detail set to 0.05 mm and the gap closing distance set to 0.1 mm without any offset) and a smoothing function (20 iterations, 0.2 smooth factor, and with shrinkage compensation enabled). These newly created models were imported into 3-matic software (Materialise, Leuven, Belgium; version 19.0), where two curves were created—one to separate the dentin and enamel surfaces from one another and the other to isolate the surface contact area of enamel—which were then used to create a separate model for the dentin and enamel surface areas. The resultant surface areas of these models were assumed to be equivalent to the surface contact area for each material (Figure 3). Surface scanning confirmed that enamel reduction had been performed to the required extent, leaving clearly exposed enamel and dentin surfaces.

### 2.4. Specimen Surface 3D Roughness Measurements

Three tooth samples were used to measure the roughness of enamel and dentin with a Mitutoyo (AVANT 3D surface roughness measuring machine) equipped with an S-3000 roughness detector module, a high-accuracy *Y*-axis table, and an auto-leveling table. The S-3000 roughness detector module consisted of a 0.75 mN detector and a standard stylus 12AAC731 with a cone angle of 60° and a tip radius of 2 µm. The measurement results were post-processed using 3D surface analysis software MCube Map Ultimate 10.3. Measurements were collected at spatial resolutions of 0.5 µm in the *X*-axis and 4 µm in the *Y*-axis, yielding 500,000 data points per cm^2^.

**Figure 4:**
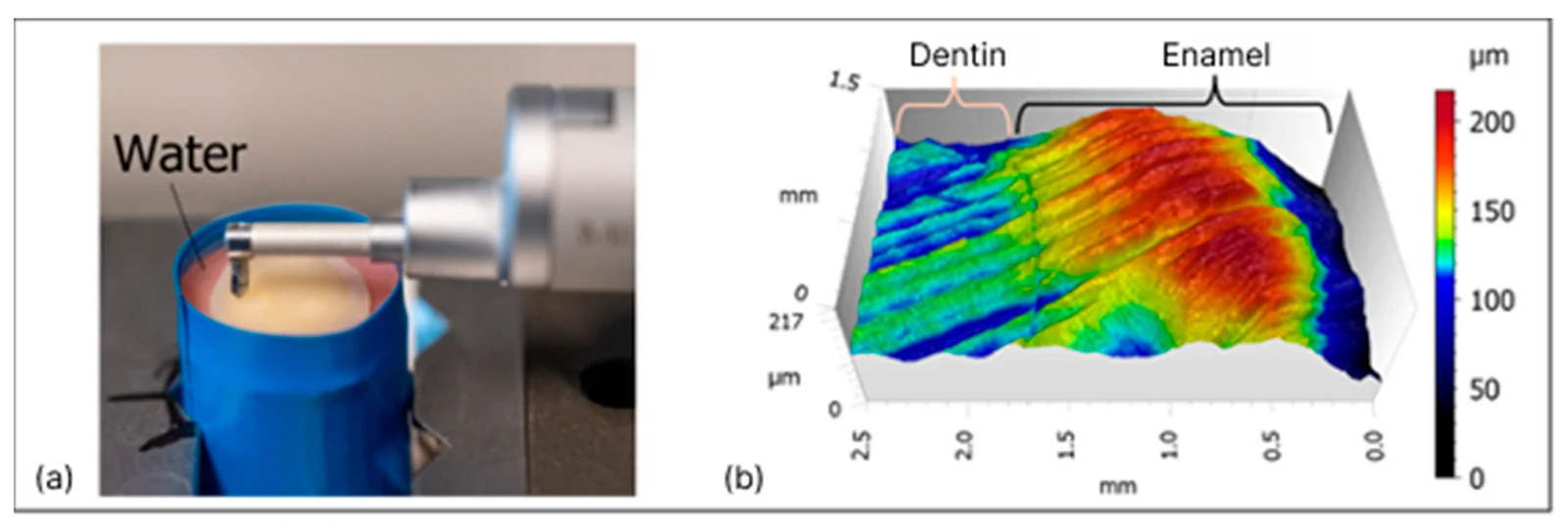
Specimen surface roughness measurement process. (**a**) The prepared samples were submerged in water to prevent them from drying out during the measurement with a profilometer. (**b**) Measured 3D surface example.

To prevent specimen dehydration, thin walls were created around each sample to function as a water reservoir, ensuring complete submersion of the tooth during measurements (Figure 4a). Preliminary testing verified that the profilometer stylus tip did not mechanically alter the tooth surface.

Surface roughness at the same location and with identical measurement settings was recorded three times:

1. Before any surface treatment,
2. After 15 s etching with phosphoric acid (K-Etchant Syringe, Kuraray Noritake)
3. After pretreatment with the Katana Cleaner (Kuraray Noritake).

Obtained 3D roughness measurements, providing visual (see example in Figure 4) and quantitative data of roughness changes after each treatment step.

### 2.5. Manufacturing of Overlays

The Ceramill Mind (Amann Girrbach AG, Koblach, Austria) was used to design 20 zirconia cylindrical-shaped overlays (Katana Zirconia YML, Kuraray Noritake, Tokyo, Japan); the diameter of each was 13.2 mm, and the height was 3.2 mm, also considering the potential shrinkage during sintering [26]. The manufacturing of the zirconia overlays was carried out following the manufacturer’s protocol for crystallization and firing [27].

### 2.6. Adhesive Cementation

Before cementation, tooth samples were divided into two groups, where the samples were provided with different cementation pre-treatments. The samples of both groups (*n* = 20) were polished with pumice DetartrineZ (Septodont), then all samples were sequentially treated with phosphoric acid K-Etchant Syringe (Kuraray Noritake) for 15 s. For one half of the samples (*n* = 10), Katana Cleaner (Kuraray Noritake) was used as an additional pre-treatment procedure. For the test group, Katana Cleaner was applied according to the manufacturer’s instructions. The prepared tooth surface was rubbed for 10 s using an applicator saturated with Katana Cleaner. The surface was subsequently treated according to the manufacturer’s recommended protocol before cementation [22]. Table 1 shows the composition of materials used in this study.

**Figure 5:**
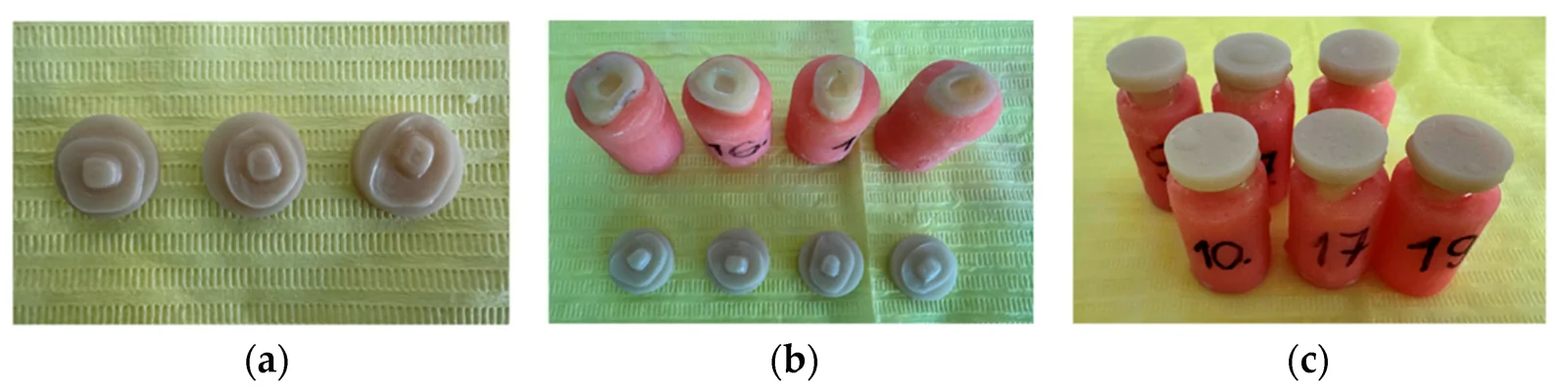
Specimen cementation flow. (**a**) Zirconia overlays after sandblasting. (**b**) Zirconia overlays prepared before cementation with checked accuracy and restoration adaptation. (**c**) Specimens after cementation.

The zirconia overlays were sandblasted (AlO_3_ 50 μm at a pressure of 2 Ba) for 15 s at a distance of 10 mm [28,29,30] and subsequently cemented with the Panavia SA Cement Universal (Kuraray Noritake, Tokyo, Japan) system following the manufacturer’s instructions [31] (Figure 5). All specimens were cemented by the same operator using consistent finger pressure to minimize variability in the seating pressure. The same cementation procedure was applied to all specimens in both groups, followed by the use of a “tack-cure” method for polymerization [25]. Afterward, the specimens were kept in a humid environment for a week [32].

### 2.7. Thermocycling

Thermocycling (Thermocycler 1100/1200, SD Mechatronik, Feldkirchen-Westerham, Germany) was carried out for a total of 5000 cycles. Each cycle lasted 60 s and consisted of 20 s in two separate distilled water baths at 5 °C and 55 °C, respectively, with a transfer time of 10 s between the baths [33].

### 2.8. Tensile Bond Strength Test

**Figure 6:**
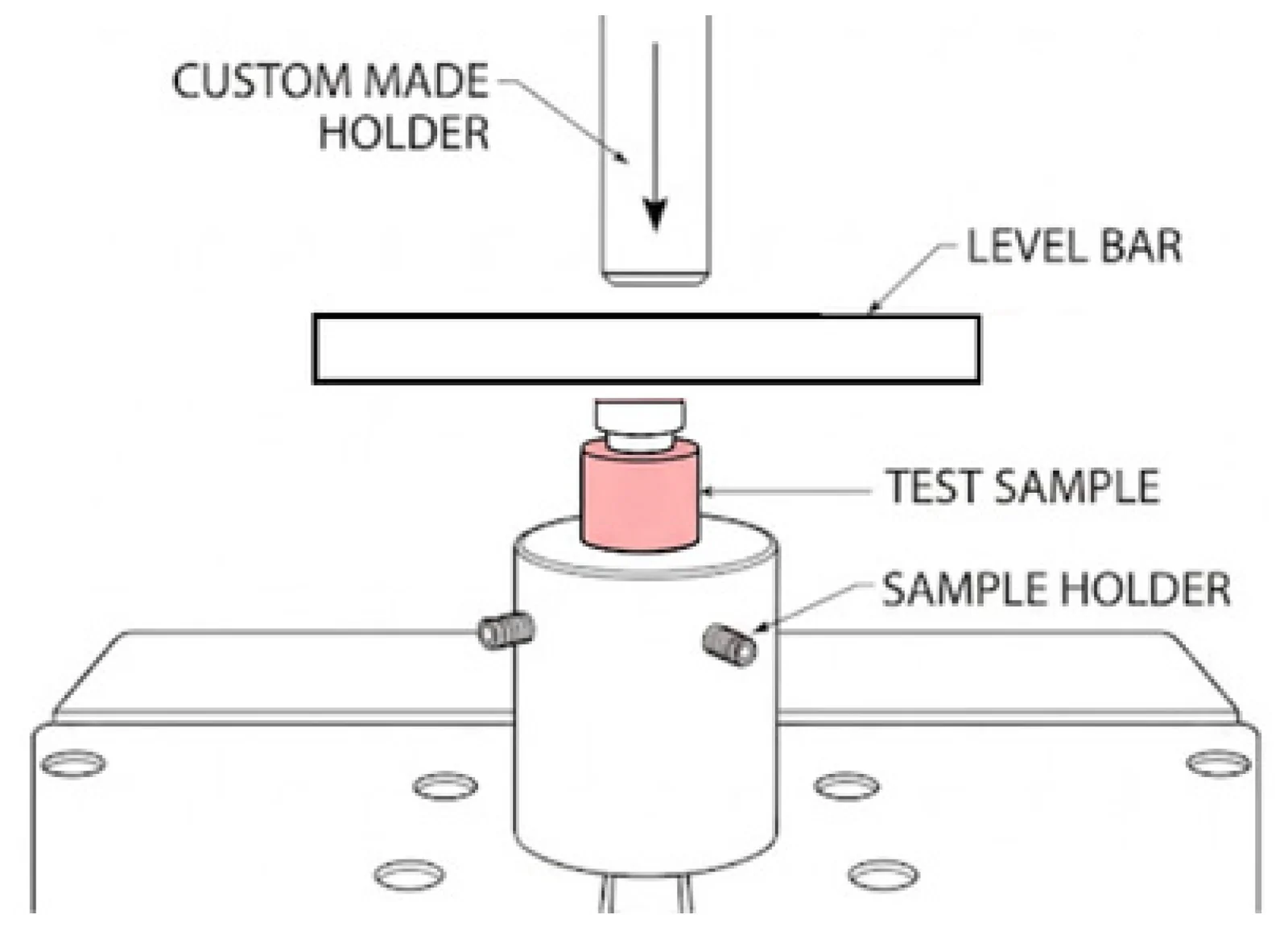
Schematic illustration of a centered and leveled specimen in a customized holder before tensile bond strength test using an Instron Universal Testing Machine.

The tensile bond strength test measured the bond strength, employing the Instron Universal Machine (Instron^®^ Mechanical Testing Systems, Norwood, MA, USA), where the head speed was set at 0.5 mm/min [ISO/TS 11405:2023] [34]. Each specimen was mounted in the testing machine using a custom-made holder which was centered and leveled to ensure that the force was evenly applied. The load at failure was indicated in Newtons (N). The test was performed blindly, meaning that the operator did not know to which study group any of the specimens belonged Figure 6.

### 2.9. Failure Mode Analysis

**Figure 7:**
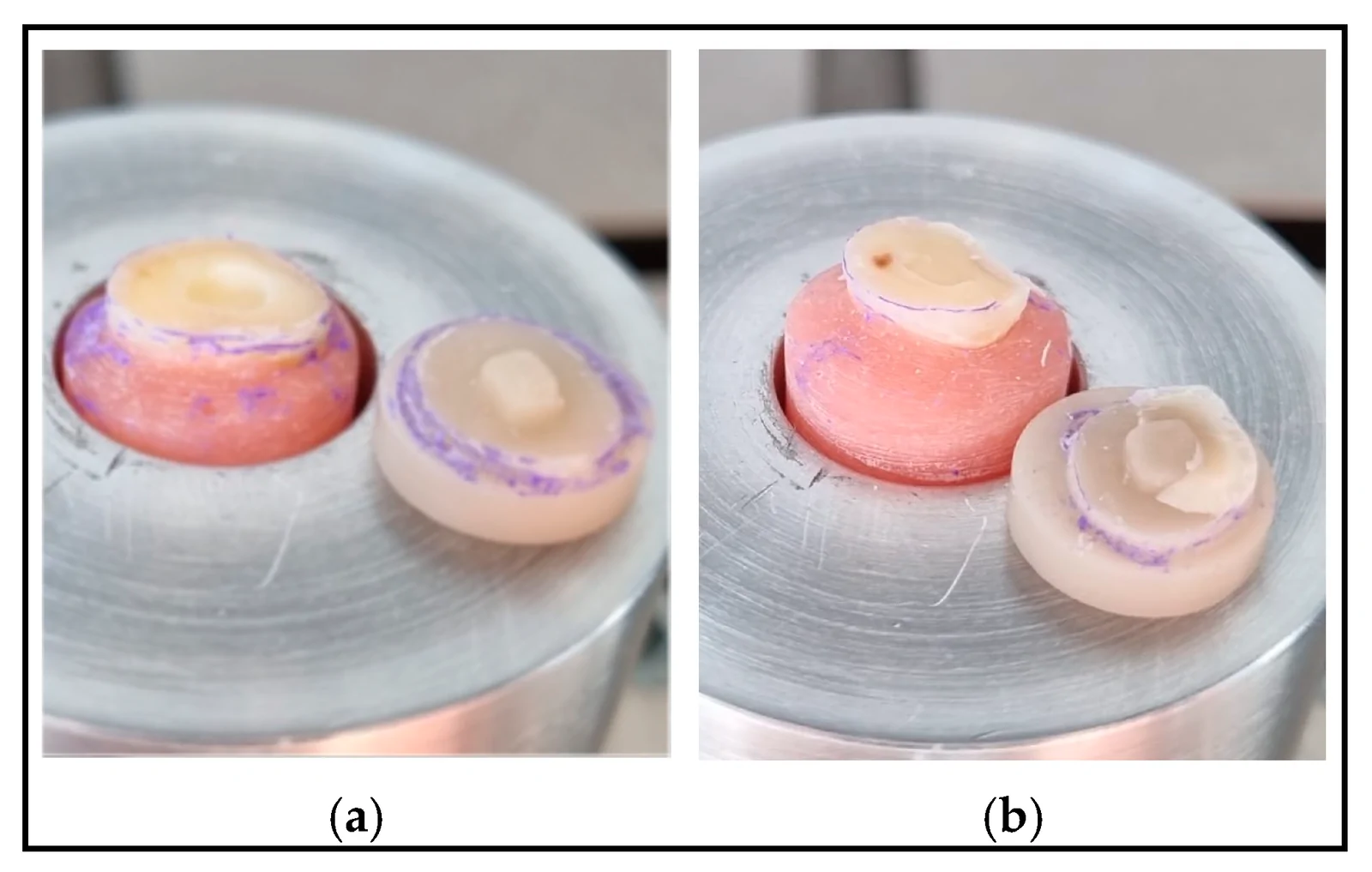
Different failure modes. (**a**) Adhesive fracture: the overlay separated from the tooth evenly, with cement on the overlay or tooth, without any visible fractures; (**b**) Cohesive fracture: the failure includes a total specimen fracture or cohesive failure, with cement present on both surfaces of the overlay and tooth specimen.

After completion of the tensile bond strength test, the specimens, including both the tooth and overlay surfaces, were qualitatively evaluated under 4× magnification using dental loupes (Orasoptic, Madison, WI, USA). Failure modes were classified as either adhesive or cohesive. Adhesive failure occurs when cement separates cleanly from the substrate surface, meaning the bond itself gave way. Cohesive failure happens when the cement splits or fractures within its own bulk, meaning the adhesive material itself broke while sticking strongly to both surfaces (Figure 7).

### 2.10. Statistical Analysis

The collected data were assessed for normality through a combination of Q-Q plots, the Shapiro–Wilk test, and the Lilliefors test. An independent two-sample *t*-test was applied to compare all variables between the two groups. Two linear regression models were made to predict the failure force for the specimens. The first model included (1) the type of specimen (test or control), (2) dentin surface area, and (3) enamel surface area, while the second model included (1) the type of specimen and (2) total tooth contact surface area. The level of significance for all tests was set to α = 0.05. All statistical computations were performed using R (version 4.3.2).

## 3. Results

### 3.1. Roughness

**Figure 8:**
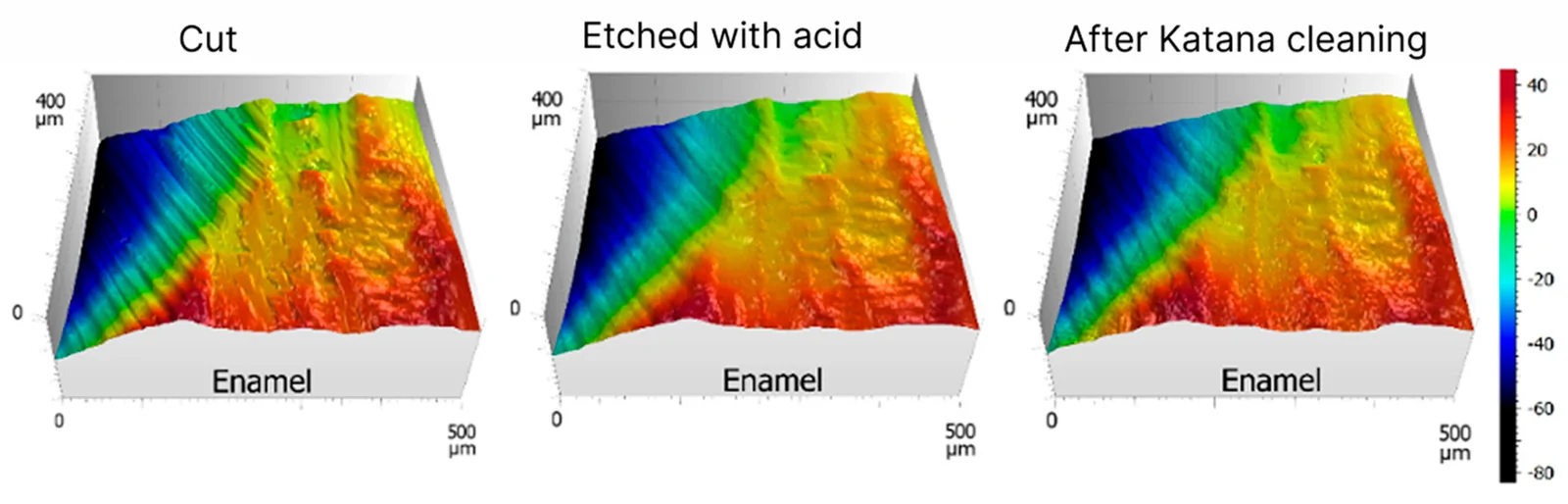
Measured 3D roughness area of sample 2 after all processing stages: cutting, etching, and cleaning. The image indicates the demineralization of sharp edges with each step.

**Figure 9:**
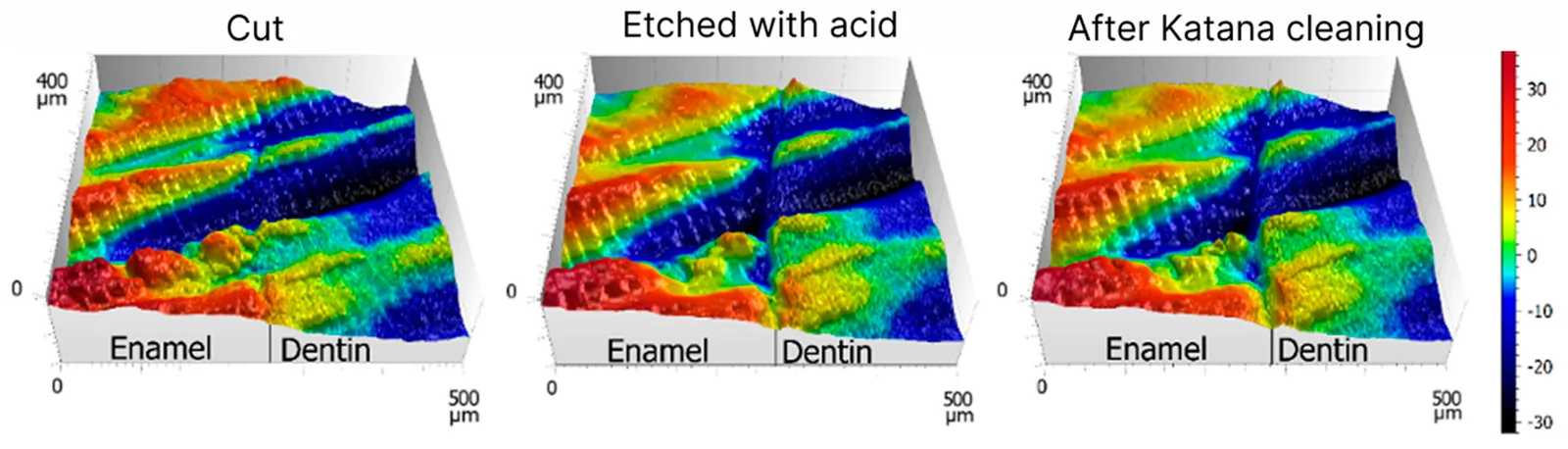
Measured 3D texture area of sample 3 after all processing stages: cutting, etching, and cleaning. The image indicates the demineralization of sharp edges and the boundary layer between the enamel and dentin with each step.

Surface 3D roughness measurements clearly indicated that etching with phosphoric acid erodes sharp edges and spikes formed during the cutting process, resulting in a surface with reduced surface area compared to the unetched surface. This is also confirmed by calculated fractal dimension values, which show that etching reduces the surface complexity. This effect was visible in all three test samples, and the etched surface affected the tooth’s enamel and dentin with moderately different intensities. Additional treatment with the Katana Cleaner continued the erosion of the tooth substance (Figure 8 and Figure 9). Acid and Katana Cleaner also significantly eroded the boundary layer between the enamel and dentin (Figure 9).

Multiple 3D roughness characterization parameters were calculated for the measured surfaces and used to quantify the visual observations. After careful analysis, the developed surface area (Adev) was considered the most suitable parameter to quantify the surface changes in this specific case. The developed surface area (Adev) quantifies the real area of the obtained surface, thus allowing us to see if the overall area of the tooth increases or decreases after etching and cleaning. The following table shows the obtained surface areas and the difference in area from the initially cut surface. Results show that etching with acid reduced the real surface by 1 to 1.5%, while further cleaning with Katana cleaner continued the area reduction by 0.1%. See Table 2.

### 3.2. Influence of Tooth Area on Bond Strength

Comparisons using unpaired *t*-tests between the two groups of specimens that were successfully tested until failure showed no statistically significant differences in any of the measured variables—failure force (*p* = 0.7205), dentin surface area (*p* = 0.3164), and enamel surface area (*p* = 0.1708). The difference in failure force between the two groups corresponded to a small effect size (Cohen’s *d* = 0.16), and a post hoc power analysis indicated very low statistical power (1 − β = 0.064). Linear regression models incorporating specimen type, dentin surface area, and enamel surface area were used to predict failure force, yielding only a modest model fit (adjusted R^2^ = 0.17, *p* = 0.1169), where dentin and enamel surface areas were the primary predictive factors. A second model in which these contact surface areas were combined into a single predictor representing total contact surface area resulted in a slightly improved fit (adjusted R^2^ = 0.22, *p* = 0.0478), indicating that specimen geometry, rather than surface treatment, dominates the prediction of failure force under the present experimental conditions.

### 3.3. Failure Modes

During the tensile bond strength test, the specimens failed differently. In the control group, 60% of the specimens exhibited cohesive failures and 40% adhesive failures. In the test group, both types of failure were also observed; however, cohesive failures predominated (90%), while adhesive failures accounted for 10%. (Table 3).

However, according to Fisher’s Exact test, no statistically significant proportional difference was observed between the two experimental groups regarding failure rates (*p* = 0.3034). The absence of statistical significance may be attributable to the limited sample sizes of the groups.

## 4. Discussion

The findings of this in vitro study did not confirm the research hypothesis that tooth surface pretreatment with Katana Cleaner (Kurary Noritake) would improve bond strength between zirconia, self-adhesive resin cement, and tooth substrate. Similar results have been reported in previous studies, suggesting that the effectiveness of zirconia cleaning agents is highly dependent on material properties and clinical conditions, particularly the presence of contamination, such as saliva and blood, which is excluded under laboratory conditions [20,35].

One possible explanation for the outcome in the present study is the chemical composition of the self-adhesive resin cement used in the study. Panavia SA (Kurary Noritake) contains functional acidic monomers such as MDP, which can form stable chemical bonds with zirconia surfaces without additional pretreatment [2,12]. Under controlled laboratory conditions, where saliva and other contaminants such as blood were eliminated, the benefit of Katana Cleaner (Kurary Noritake) may therefore have been limited. The assumption is that surface decontamination agents play a more critical role in clinical situations where contamination is unavoidable, which is a common cause of cementation failure in everyday practice [36] and is something that may be evaluated in future studies. The self-adhesive cement system Panavia SA (Kurary Noritake) itself has potential and may perform differently compared to cement systems without MDP, providing improved bonding [37].

Katana Cleaner was initially developed for the cleaning of zirconia ceramic surfaces and contains a 10-methacryloxydecyl dihydrogen phosphate (10-MDP) salt derived from the phosphate ester resin monomer MDP, together with triethanolamine. In addition to its cleaning action, the 10-MDP-containing formulation exhibits surface-active properties that facilitate the removal of organic contaminants from the substrate and their subsequent removal by water rinsing. The presence of 10-MDP also provides the potential for chemical interaction with mineralized dental substrates, including zirconia, dentin, and enamel, and may therefore influence subsequent bonding interactions. However, the extent and clinical relevance of this potential effect may depend on the specific substrate, surface condition, and adhesive or cement system used. With a pH of approximately 4.5, Katana Cleaner is formulated for both intraoral and extraoral use. Unlike strongly alkaline cleaning agents such as sodium hydroxide (NaOH) and potassium hydroxide (KOH), Katana Cleaner is designed to be compatible with both tooth structures and prosthetic surfaces, making it a versatile cleaning agent for dental applications [22,38].

Previous studies have reported that the use of Katana Cleaner on contaminated dentin may improve the bond strength of self-adhesive resin cements compared with water rinsing alone, while other studies have demonstrated improved or restored bonding to contaminated zirconia surfaces. These effects have been partly attributed to the cleaning action of the material and the potential chemical reactivity of its 10-MDP component with mineralized substrates. However, the available evidence remains inconclusive, as recent studies have not consistently demonstrated a significant improvement in immediate or long-term bond strength following the use of 10-MDP-containing cleaners. Although the difference in bond strength between the groups in our study did not reach statistical significance, the Katana Cleaner group demonstrated a noticeably higher proportion of cohesive failures. This finding may indicate a more favorable bonding performance following Katana Cleaner application; however, it should be interpreted cautiously given the limited sample size and the absence of a statistically significant difference in bond strength [21,39].

Beyond chemical bonding, cavity retention factors may play a particularly important role. The combination of preparation design with sufficient cavity retention and a larger bonding surface area may be more decisive for bond strength than surface pretreatment alone. This assumption is supported by the finding that specimens with larger tooth surface areas demonstrated higher bond strength values when cemented with Panavia SA (Kurary Noritake). In this context, self-adhesive cements appear to perform more predictably when both retention cavity and adequate bonding area are present. Similar outcomes were observed in the study conducted by Guilardi [12].

Surface roughness analysis revealed an apparent decrease in measured roughness after each pretreatment step, which contrasts with the established concept that acid etching increases surface roughness and micromechanical retention [40]. This discrepancy can be explained by methodological limitations of contact profilometry, as the stylus tip is unable to detect micro- and nano-scale changes within dentinal tubules. Consequently, the recorded values reflect alterations of the outer surface layer rather than the true increase in internal surface area. Nevertheless, the observed surface changes indicate that pretreatment procedures do modify tooth substrates and may influence bonding behavior. An important strength of this study is the use of micro-CT scanning to assess the tooth surfaces. This method allowed for precise determination of the prepared surface area as well as accurate identification of the substrate, distinguishing between enamel and dentin. Visual assessment alone is not sufficiently reliable for determining whether enamel has been removed during preparation. Therefore, the application of micro-CT enhanced the accuracy of measurements and improved the overall methodological quality of the study.

Because measurable surface alterations were observed after phosphoric acid and Katana Cleaner application, it was clinically relevant to investigate whether these changes could influence the cementation process. Katana Cleaner (Kurary Noritake) was included in this study because a clean and properly prepared tooth surface is considered essential for achieving reliable adhesion during indirect restoration cementation. Its intended role is to remove organic contaminants and optimize the substrate before bonding, potentially improving the interaction between the tooth surface, resin cement, and zirconia restoration. The broad clinical strategy of Katana Cleaner also contributed to its selection for investigation, as the material can be used both on the restoration surface and intraorally for cleaning the tooth surface, providing versatility in clinical application.

The choice of molars as test specimens further supports this interpretation. Clinically, molars are often extensively damaged and therefore require indirect restorations with larger bonding surfaces. At the same time, posterior teeth present significant challenges in terms of access and isolation, making technique-sensitive multi-step adhesive protocols difficult to apply consistently. Self-adhesive resin cements may therefore represent a practical compromise between simplified clinical procedures and acceptable long-term bonding reliability, particularly in posterior teeth [41]. However, the results also indicate that the suitability of self-adhesive cements is case-dependent, as their performance may be less predictable in situations with limited bonding surfaces or minimally retentive preparations [41,42].

The use of overlay restorations was selected because this design reflects a contemporary minimally invasive approach for restoring endodontically treated posterior teeth. By preserving more remaining tooth structure than full-coverage crowns, overlays may reduce further weakening of already compromised teeth while still providing cuspal protection. In such restorations, the interaction between restoration design, bonding surface area, and cement properties becomes particularly important for long-term success [43,44].

Thermocycling was applied to simulate long-term aging and to evaluate the durability of the adhesive interface under conditions closer to the oral environment. Thermal stresses caused by repeated temperature changes are known to contribute to bond degradation and restoration failure [45]. The inclusion of thermocycling therefore allowed differentiation between immediate bond strength and more clinically relevant long-term performance. Previous studies have shown that self-adhesive cements may demonstrate satisfactory initial adhesion but can be susceptible to hydrolytic and thermal degradation over time, particularly at the dentin interface [29,46]. Nevertheless, evidence remains heterogeneous. Systematic reviews suggest that although immediate bond strength is often satisfactory, long-term durability following thermocycling or water storage may be material-dependent and sometimes inferior to multi-step systems [47,48]. Therefore, SARCs may not be equally suitable in all clinical situations, particularly where bonding surfaces are limited, or mechanical stresses are high.

Failure mode analysis provided additional insight into the bonding mechanism. A higher proportion of cohesive failures was observed in the Katana Cleaner group compared with the untreated group. This may indicate a stronger adhesive interface in the cleaned specimens, even though no statistically significant difference in bond strength values was detected. These findings suggest that Katana Cleaner may influence the quality of the bonded interface in a way that is not fully reflected by tensile bond strength alone; however, it should be interpreted cautiously given the limited sample size and the absence of a statistically significant difference in bond strength.

The in vitro nature of the present study represents a limitation, as the experimental conditions cannot fully reproduce the complex oral environment, including variations in saliva, masticatory forces, pH fluctuations, enzymatic activity, and thermal and mechanical stresses.

Although thermocycling was used to simulate thermal aging, the number of cycles and experimental conditions cannot fully reproduce the long-term intraoral aging process.

Regarding mechanical testing, tensile bond strength testing provided controlled and reproducible measurements but did not fully replicate the complex loading conditions of the oral environment, where shear and compressive forces predominate [49]. Although tensile testing offered valuable comparative data, future studies combining tensile, shear, and fatigue testing could provide a more comprehensive understanding of the clinical performance of self-adhesive resin cements.

The main limitation of this study is the small sample size (n = 10 per group), which limits the statistical power and generalizability of the results. Therefore, the findings should be considered preliminary, and studies with larger sample sizes are needed for more reliable conclusions. Additionally, variability among teeth, including differences in surface area and enamel-to-dentin ratio, as well as minor inconsistencies arising from manual preparation despite standardized protocols, may have influenced the results.

A limitation of the present study is that no formal sample size calculation was performed. The sample size was determined based on the availability of intact third molars, the feasibility of specimen preparation, and the practical constraints of the study, while considering sample sizes commonly used in previous in vitro studies. Although this approach was considered feasible for the experimental design, the absence of a formal power analysis may have limited the statistical power to detect smaller differences between the groups. Therefore, the findings should be interpreted with consideration of the relatively limited sample size.

Only one type of restoration, one type of restorative material, and one type of cement -self-adhesive resin cement—was used to isolate and evaluate the specific effect of Katana Cleaner without introducing additional variability into the study data. It could be considered that a limitation of this study is the restricted selection of materials and treatment design, which may limit the applicability of the findings to other restorative approaches. Therefore, future studies comparing different materials, restoration designs, and cement types are necessary to provide a more comprehensive understanding of clinical performance.

Another limitation of the present study is that a standardized cementation load was not applied using a calibrated device. Although all specimens were cemented by the same operator using a consistent finger-pressure technique to minimize variability, the applied force could not be precisely quantified or controlled. Therefore, some variation in cementation pressure between specimens cannot be excluded and may have contributed to variability in the measured bond strength. Future studies should consider the use of a calibrated loading device to standardize the cementation force more precisely.

Further studies involving larger sample sizes are recommended to confirm the present findings. Prospective long-term clinical studies would also be valuable to determine whether the observed effects translate into improved clinical performance and remain stable over several years.

Overall, the findings suggest that in self-adhesive cementation, mechanical retention and bonding surface area may play a more critical role than surface pretreatment under ideal laboratory conditions, while surface decontamination may become more important in clinically contaminated environments.

## 5. Conclusions

Within the limitations of this in vitro study, considering the small sample size and limited statistical power, results suggest that bond strength of zirconia overlays cemented with self-adhesive resin cement is primarily influenced by the size of the dental substrate area and the presence of adequate cavity retention, while additional surface pretreatment in the form of a cleaning agent showed no significant effect under controlled conditions. Further studies with larger sample sizes are recommended to confirm the findings.

## Figures and Tables

**Table 1:** Materials used, their corresponding composition and manufacturer.

Material	Manufacturer	Composition
KATANA Cleaner	Kuraray Noritake Dental Inc., Tokyo, Japan	Water; 10-MDP; polyethylene glycol; triethanolamine; stabilizer, dyes.
PANAVIA SA Cement Universal	Kuraray Noritake Dental Inc., Tokyo, Japan	Paste A: 10-MDP, bisphenol A diglycidylmethacrylate (Bis-GMA), triethyleneglycol dimethacrylate (TEGDMA), hydrophobic aromatic dimethacrylate, 2-hydroxyethyl methacrylate (HEMA), silanated barium glass filler, silanated colloidal silica, potassium persulfate, dl-camphorquinone, peroxide, catalysts, and pigments. Paste B: hydrophobic aromatic dimethacrylate, silane coupling agent, silanated barium glass filler, aluminum oxide filler, surface-treated sodium fluoride, dl-camphorquinone, accelerators, and silanated titanium dioxide.
KATANA Zirconia YML	Kuraray Noritake Dental Inc., Tokyo, Japan	ZrO_2_ (80–95%), Y_2_O_3_ (3–15%)
K-Etchant Syringe	Kuraray Noritake Dental Inc., Tokyo, Japan	Phosphoric acid, polyethylene glycol, colloidal silica, water, and pigment.

**Table 2:** Measured surface areas.

Developed Area, mm^2^					
Sample	Cut	Etched	Area Reduction, %	Cleaned	Area Reduction, %
Sample 1	1.245	1.231	−1.1	1.229	−1.2
Sample 2	4.846	4.774	−1.5	4.773	−1.5
Sample 3	3.982	3.923	−1.5	3.918	−1.6

**Table 3:** Distribution of failure modes in the control and test—Katana Cleaner groups.

Group	Cohesive Failures, n (%)	Adhesive Failures, n (%)	Total, n
Control group	6 (60%)	4 (40%)	10
KATANA Cleaner group	9 (90%)	1 (10%)	10
Total	15 (75%)	5 (25%)	20

## Data Availability

The data that support the findings of this study are available from the corresponding author, S. Grinberga, upon request.
